# Another Page Turns for Journal of Neonatal Surgery

**Published:** 2016-01-01

**Authors:** Yogesh Kumar Sarin

**Affiliations:** Department of Pediatric Surgery, Maulana Azad Medical College, University of Delhi, New Delhi, India

To start, and pursue a journal dedicated to a superspecialty with meager monetary and human resources has not been an easy feat. On expected lines, we mainly received case reports in its infancy. Slowly, we attained acceptance globally and the authors from all the continents started contributing. We fulfilled our promise to archive every manuscript on PubMed Central that we have published since the inception of JNS in 2015. It has been a learning opportunity for us as well. We take this opportunity to express our gratitude to the authors who trusted the journal and its editorial team. Though we struggle to get original research papers and random controlled trials, we have strived to maintain the standards of the contents and honored the publication dates. Aged four, we are presently full of energy, curious and eager to show what we can do. The stage of waffling between feelings of security and insecurity is gone. We are excited by our accomplishments. We are looking ahead to get indexed with Medline and official Impact Factor.

## Footnotes

**Source of Support:** Nil

**Conflict of Interest:** The author is editor of the journal. This article is not externally peer reviewed.

